# Assessment of embryotoxic effects of quinoline yellow using attention-based convolutional neural network and machine learning in zebrafish model

**DOI:** 10.3389/fphar.2025.1606214

**Published:** 2025-08-01

**Authors:** Magdalena Majdan, Piotr S. Maciąg, Agata Rogalska

**Affiliations:** ^1^ Department of Toxicology and Food Science, Faculty of Pharmacy, Medical University of Warsaw, Warsaw, Poland; ^2^ Warsaw University of Technology, Faculty of Electronics and Information Technology, Institute of Computer Science, Warsaw, Poland

**Keywords:** embryotoxicity, zebrafish, quinoline yellow, convolutional neural network, transfer learning

## Abstract

Our daily diet often includes food additives found in numerous processed foods. Growing concerns about the toxicity and potential health risks of synthetic dyes have drawn increased attention from researchers and regulatory authorities. This study examines the embryotoxic effects of Quinoline Yellow (QY), a synthetic dye commonly used as an additive, using both *in silico* and *in vivo* models. Computational studies on QY were conducted using QSAR (Quantitative Structure Activity Relations) analysis to identify the major toxicological endpoints. *In silico* predictions indicated clastogenic and reproductive toxicities, interaction with androgen and estrogen receptors, and an elevated propensity for skin and respiratory allergies. *Danio rerio* (zebrafish) embryos were exposed to various concentrations of QY (0.005–2 mg⋅mL^−1^) over 48, 72 and 96-h periods. Lethal effects were observed at concentrations above 0.5 mg mL^−1^, with a median lethal concentration LC50 of 0.64 mg mL^−1^. Exposure to QY (0.5–2 mg⋅mL^−1^) resulted in pericardial edema, swollen and necrosed yolk sac, blood stasis and reduced eye size. The study provides direct evidence for the developmental toxicity and teratogenic potential of QY. To enhance the analysis, attention-based Convolutional Neural Networks (CNN) and Transfer Learning (TL) were employed to discern morphological alterations in zebrafish embryos exposed and not exposed to QY. Automating the analysis and classification of zebrafish embryo images diminishes the workload and time burden on biological experts while simultaneously enhancing the reproducibility and objectivity of the classification. The developed neural network further corroborates the evidence suggesting QY’s potential toxicity.

## Graphical Abstract

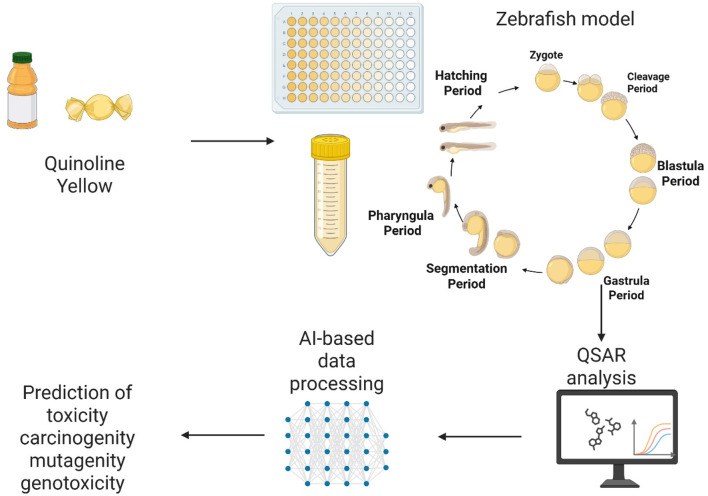

## 1 Introduction

Recent epidemiological studies have shown a strong correlation between consuming Ultra-Processed Foods (UPFs) and an increased risk of developing various chronic diseases (Jardim et al., 2021). The use of artificial dyes in the industry remains a topic of considerable controversy and debate among both scientists and consumers (Rambler et al., 2022; Amchova et al., 2015; Debras et al., 2022). While food additives at acceptable levels are deemed safe for human consumption, their widespread presence and conflicting data suggest the potential for toxicological risks to both humans and marine organisms (Tkaczyk et al., 2020; Li et al., 2024). The ADI, expressed in mg/kg/day, represents the maximum amount of a substance a person can ingest daily throughout their lifetime without causing harm. Estimating the adequate ADI level is challenging, especially since many consumed products contain the same additives. It is widely acknowledged that children are the demographic most at risk of exceeding the recommended daily intake of food additives. Numerous food additives are found in products that are particularly popular among children, such as sweets, flavoured drinks, ice cream, sausages, and fast food. Furthermore, there is a lack of conclusive evidence regarding the impact of food additives, environmental contaminants, cosmetic ingredients, and pharmaceuticals during the prenatal period and early childhood. Due to numerous reports of adverse effects on consumers, the list of synthetic colours is being systematically updated (Amchova et al., 2015; Østergaard and Knudsen, 1998; Soni et al., 2024). On the other hand, data on the exposure of aquatic organisms to colourings is lacking in the world literature. Children are at greater risk of developing chronic diseases resulting from early exposure to environmental substances. However, with access to advanced toxicity prediction methods and new test models (zebrafish), safety studies of synthetic dyes are warranted.

QY belongs to the group of quinophthalone dyes. Commercially available as a mixture of monosulphonic, disulphonic, and trisulphonic acid derivatives, QY is used in confectionery, isotonic, and carbonated drinks. It can cause allergies and hyperactivity in children, as well as potentially be mutagenic and genotoxic (Chequer et al., 2015; Chequer et al., 2017; McCann et al., 2007; Scientifc Opinion on the re-evaluation of Quinoline Yellow, 2009). On the other hand, a study conducted in rats where QY was administered subcutaneously did not demonstrate evidence of carcinogenicity. The available *in vivo* oldest evidence does not indicate a genotoxic effect of quinoline yellow. Several methods have been used in *in vitro* and *in vivo* testing, including bacterial mutation assays, the L5178Y mouse lymphoma gene mutation assay, and the NMRI mouse micronucleus assay (Scientifc Opinion on the re-evaluation of Quinoline Yellow, 2009). In 2009, the European Food Safety Authority (EFSA, 2025; Scientifc Opinion on the re-evaluation of Quinoline Yellow, 2009) panel reduced the Acceptable Daily Intake (ADI) of QY from 10 mg/kg/b.w. to 0.5 mg/kg/b.w. A study examining the impact of the combination of food colours known as ‚Southampton’ (Tartrazine, Quinoline Yellow, Sunset Yellow, Ponceau 4R, Allura Red AC, Carmoisine) and Sodium Benzoate revealed an augmentation in behavioural activity among children (McCann et al., 2007). Nevertheless, there is a certain degree of ambiguity in interpreting the outcomes of these subsequent tests when evaluating the safety of quinoline yellow. This discrepancy can be attributed to the fact that the studies above used a test substance containing a high proportion of the monosulphonate component, ranging from 85% to 91%. In contrast, the specifications for QY intended for food use indicate that disulphonate is the primary component (over 80%), with monosulphonate present only in minimal amounts (15%). These compositional differences could significantly affect the safety assessment, highlighting the need for further research. Recent studies by Macioszek and Kononowicz (Macioszek et al., 2004) suggest that QY may have clastogenic and/or mutagenic properties, potentially causing DNA damage. An additional concern is the widespread use of QY in pharmaceuticals and cosmetic products. Although regulatory guidelines set permissible levels of synthetic dyes in individual products, they often fail to account for cumulative exposure, including that originating from environmental sources.

The *Danio rerio* has been demonstrated as an invaluable tool for the expedient screening of chemical toxicity, as it identifies numerous phenotypic abnormalities (McColl et al., 2011). Furthermore, *Danio rerio* retains key vertebrate traits such as high fecundity, rapid development, and translucent juveniles, all of which contribute to the speed and ease of experimentation compared to mammalian models. Despite specific anatomical and physiological differences characteristic of aquatic species, *Danio rerio* possesses most organs analogous in structure and function to those of humans (MacRae and Peterson, 2015).

Nevertheless, the primary challenge remains in the labour-intensive morphometric analysis of the acquired microscopic images, which limits the attainment of maximum throughput. The application of machine learning tools holds the potential to facilitate the development of models capable of distinguishing between the morphological characteristics of diverse organisms, including *Danio rerio* (Lin et al., 2018). Initial studies concentrated on identifying specific embryonic phenotypes, such as mortality and developmental stages. However, processing large datasets and distinguishing between numerous sublethal morphological abnormalities continue to pose significant challenges. Recent literature on *Danio rerio* has primarily focused on specific organs (Dong et al., 2023). Furthermore, researchers are interested in quantitative measurements, such as body length and curvature. Consequently, it is crucial to develop tools that can simultaneously capture the most prevalent phenotypes. The application of machine learning to replace manual measurements would undoubtedly enhance the applicability and efficiency of the zebrafish model. Convolutional Neural Networks (CNNs) are the example of such model commonly employed for image recognition due to their ability to efficiently capture spatial hierarchies in images through convolutional layers that detect edges, textures, and patterns. The CNN architecture diminishes the necessity for manual feature extraction, enabling the network to learn pertinent features directly from raw images. Moreover, CNNs’ capacity to handle extensive volumes of high-dimensional data renders them highly effective for intricate image classification tasks (Gu et al., 2018; Li et al., 2022).

Animal tests’ high costs and time-intensive nature pose significant challenges to evaluating chemicals on domestic and international markets. *In silico* techniques, such as computer modelling and analysis, offer an alternative by predicting the toxicity of chemicals based on correlations between molecular properties and biological activity. The primary advantage of *in silico* methods lies in their utility during early research stages. Virtual screening of molecular libraries facilitates the rapid identification of promising structures from thousands of potential candidates. For instance, these methods can eliminate molecules with specific structural alerts (toxicophores) that may indicate a particular toxic effect. *In silico* techniques are distinguished by their efficiency, speed, low cost, and precision, making them particularly valuable prior to synthesising chemical compounds, including potential medicinal substances (Segall and Barber, 2014). In recent years, numerous models for toxicity prediction have been developed to support risk assessment, along with open-access websites that leverage machine learning and structural alerts, all of which are freely available (Cheng et al., 2012). Quantitative Structure-Activity Relationship (QSAR) and Quantitative Structure–Toxicity Relationship (QSTR) models, which predict biological and/or physicochemical properties from the structural parameters of a chemical compound, constitute a well-established approach to chemical data analysis. They are particularly valuable for predicting various toxicity indices, including mutagenicity, carcinogenicity, and acute toxicity (Kar and Leszczynski, 2019).

This study investigated the potentially toxic effects of QY in a model of *Danio rerio* (zebrafish). The scope of the work included conducting an experiment using different concentrations of QY, assessing survival, embryo morphology, and possible developmental defects. *In silico* analysis of QY toxicity was also conducted using the ADMET Predictor software. Additionally, a machine learning protocol was established to analyze toxic endpoints in zebrafish embryos treated with QY.

## 2 Materials and methods

### 2.1 Materials

All solvents and inorganic chemicals used in this study were of analytical grade. The standard of QY (CAS number 8004-92-0) and tricain (CAS number 886-86-2) were purchased from Sigma Aldrich (Hoeilaart, Belgium). Millipore DirectQ UV3 system (Darmstadt, Germany) was used as the source of water (R > 18 MΩ cm). The zebrafish embryos (AB TL line) were sourced from the International Institute of Molecular and Cell Biology in Warsaw and maintained in E3 medium (concentrate of NaCl, KCl, CaCl_2_, MgSO_4_). Embryo staging was conducted in accordance with the criteria established by Kimmel et al. (1995).

### 2.2 Methods

#### 2.2.1 Procedure for toxicity testing

Zebrafish embryos (AB × TL) were obtained from the International Institute of Molecular and Cell Biology in Warsaw and maintained in E3 medium. The embryos were identified according to Kimmel et al. (1995), and only the fertilized ones that showed the process of cell division were selected. At 6 h post fertilization (hpf), the embryos were placed on 96-well plates (one embryo per well, twenty for one group) with previously. The embryos were treated with QY at concentrations ranging from 0.005, 0.02, 0.1, 0.5, 0.75, 1, 1.5, 2 mg mL^−1^ for 96 h post-fertilisation (hpf), and the resulting morphological changes were assessed by the guidelines outlined in OECD 236 (OECD, 2013). Observations were made at 24-h intervals up to 96 hpf. The experiment was conducted in triplicate under identical conditions. Currently, the European Commission Directive 2010/63/EU permits experimentation in fish embryos at the earliest life stages without being regulated as animal experiments; zebrafish are considered models *in vitro* until 120 hpf [http://data.europa.eu/eli/dir/2010/63/2019-06-26 (EFSA, 2025)]. The plates were incubated at a constant temperature of 27°C ± 1°C with a light-dark cycle (12 h/12 h) throughout the study period. The embryos were analyzed under a microscope (Olympus CKX53), and images were captured using an Olympus EP50 camera (CAM-EP50). The length, width of embryos and eye size were measured using EPview1.3 software (Olympus, Tokyo, Japan).) at 96 hpf. A tricaine (0.3%) solution was applied at the end of the experiment for euthanasia.

#### 2.2.2 *In silico* studies using the software ADMET Predictor

A comprehensive measure of toxicological endpoints was obtained through the calculation using the software ADMET Predictor™ version 10.1 (Simulation Plus, Lancaster, CA) and described in the Results section.

#### 2.2.3 Statistical analysis

Statistically significant differences between groups were evaluated using an ANOVA followed by the Dunnett *post hoc* test or non-parametric Kruskal-Wallis test. Statistical significance was defined as p < 0.05. Data were presented as mean ± SEM. Data analysis was with GraphPad Prism software version 8 (GraphPad Software, San Diego, United States).

### 2.3 Classifying phenotypes with neural networks

#### 2.3.1 The designed classification architecture

**FIGURE 1:**
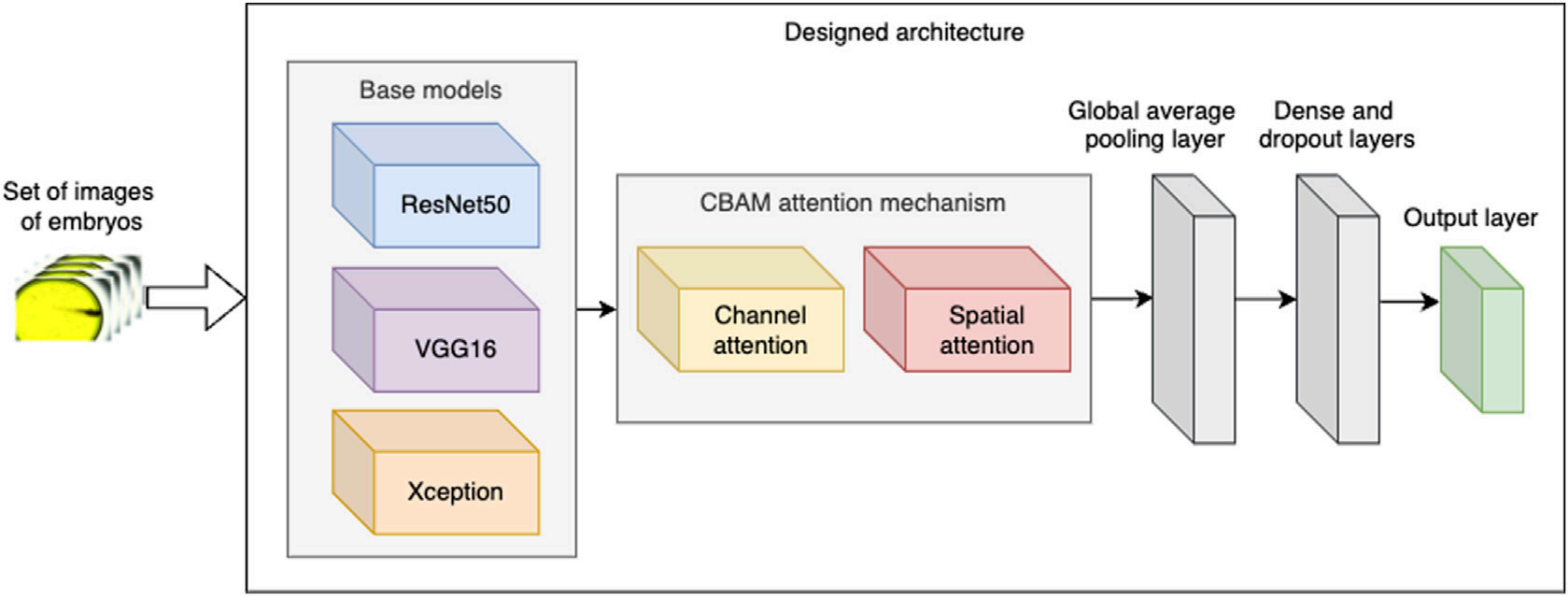
Designed architecture of the neural network.

To classify the acquired images of embryos’ phenotypes, we designed three versions of a CNN architecture, each of which was constructed by fine-tuning a pretrained base model and incorporating the Convolutional Block Attention Module (CBAM) mechanism (Figure 1). For the base models, we selected the ResNet50, VGG16, and Xception neural networks, each of which was sourced from the Keras library. ResNet50 is a deep learning model distinguished by its 50-layer architecture comprising convolutional layers with ReLU activation units, pooling layers, and batch normalisation layers (Tensorflow.keras.applications.resnet50, 2020). The incorporation of residual blocks in ResNet50 improves classification accuracy by addressing the vanishing gradient problem, a prevalent issue in deep neural networks. ResNet50 has been extensively applied in tasks such as image classification, object detection, and transfer learning.

VGG16 is another base model selected by us for the experiments. VGG16 was previously used in the experiments classifying *Danio rerio* embryos’ phenotypes (Tyagi et al., 2018).VGG16 consists of 13 convolutional layers, 3 fully connected layers, and 5 max pooling layers (Simonyan and Zisserman, 2014). The base VGG16 model was trained over one million images from the ImageNet dataset. The experiments presented by Tyagi et al. (2018) suggest that VGG16 network can provide good classification results when fine-tuned with the acquired images of *Danio rerio* phenotypes if a relatively small number of classes (e.g., five) was used in classification.

Finally, as the third base model tested in the experiments, we selected the Xception model (Chollet, 2017). The model’s architecture consists of 36 convolutional layers structured into 14 modules, all but first and last having linear residual connections around them.

Subsequently, in the designed architecture, we integrated the base model with the CBAM attention mechanism. CBAM applies attention in two stages:

• *Channel attention* aims to enhance the information conveyed by the channels of each convolutional layer by assigning a weight to each channel, which is learned during the model’s training process. Higher weights indicate greater importance of the corresponding channels.
• *Spatial attention* mechanisms concentrate on the most significant spatial locations within each channel. For our data, the spatial attention mechanism aims to (i) enhance the detection of blood stasis by focussing on blood clots and (ii) minimise noise in the input images by emphasising regions containing actual embryos.

Following the implementation of CBAM, we incorporated two additional intricate layers: global average pooling as well as a dense and dropout layer. The dense layer comprises 512 neurons, and the dropout rate is set to 0.5. These concluding layers are employed to reduce the network’s complexity and mitigate overfitting, thereby augmenting the model’s ability to generalise effectively.

#### 2.3.2 Neural Network’s training procedure

In order to train the network, we applied the following procedure:

1. The model is trained using the images of embryos provided by Jeanray et al. (2015). To this end, we utilised the learning component of the dataset described therein. For the purpose of our experiments, we selected images from five classes: Dead, Edema, Blood stasis, Necrosed yolk sac, and Normal. This selection corresponds to the categorization of embryos in our experiments on QY exposure.
2. Subsequently, the designed model is *fine-tuned* with the above-mentioned selected data of Jeanray et al. (2015). This fine-tuning process adjusts the weights of one-third of the final layers of the base model, as well as all other layers added by us.
3. Finally, the model undergoes a second round of fine-tuning using data from our experiments. This time, fine-tuning is applied to all layers of the network, including both the base model layers and the additional layers integrated into the architecture.

We provide the exact learning parameters of the model in Table 2. We opted to utilise the Jeanray et al. (2015) dataset in the training of our model for two primary reasons: (i) to augment the classification accuracy of our model, as their images exhibit a high level of structure and quality; and (ii) due to the limited size of our own collected dataset of *Danio rerio*.

#### 2.3.3 Data augumentation

The data used to train the designed architecture are augmented by applying two operations:

1. To enhance the recognition of blood stasis, we multiplied the red channel values by 1.5 to increase the intensity of the red color.
2. We added further augmentation, including image rotation, zoom, horizontal flipping, and brightness adjustments, using the *ImageDataGenerator* object from the *Keras* library.

#### 2.3.4 Preparation of datasets used for the Network’s training

**FIGURE 2:**
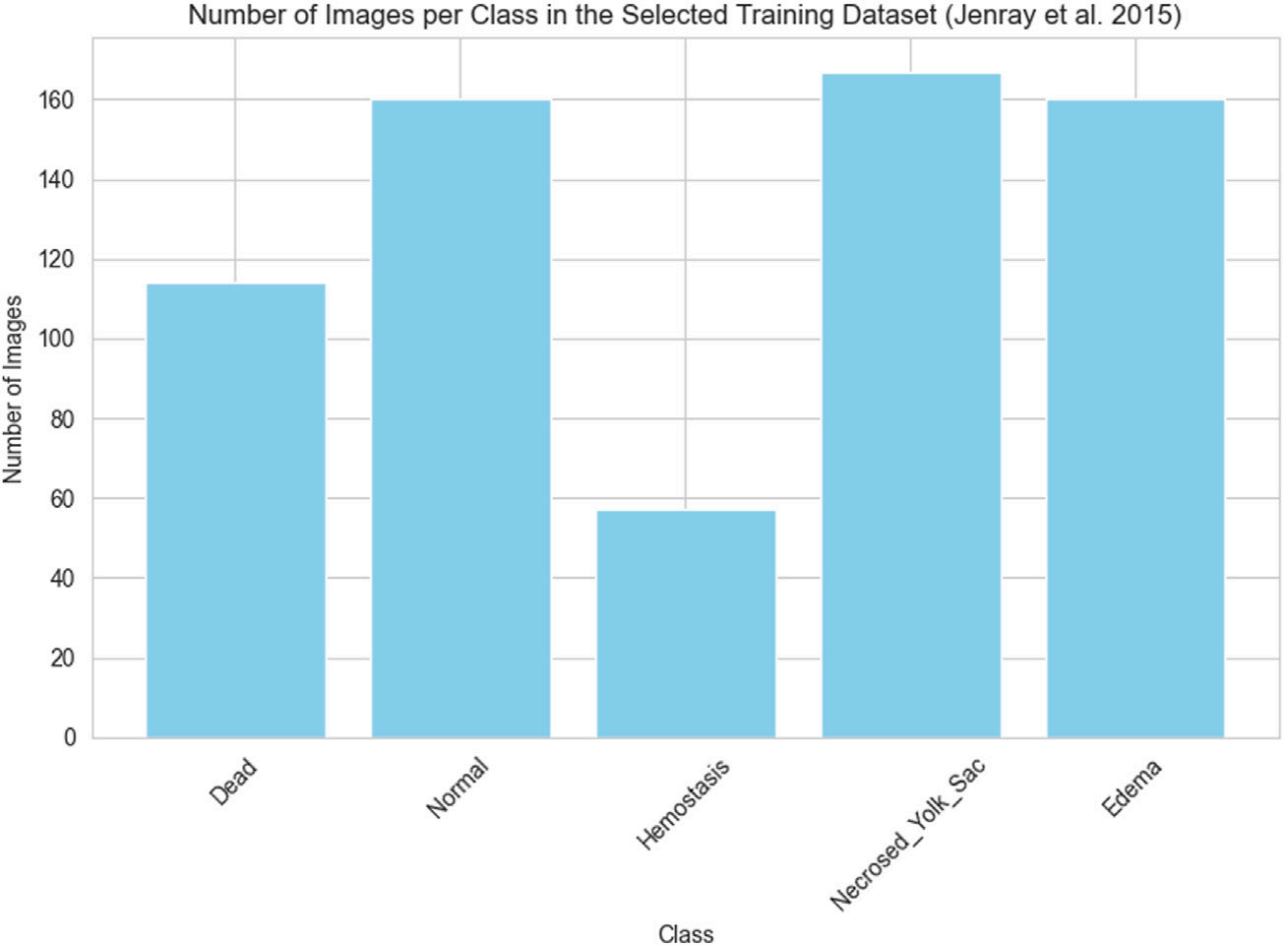
The number of images in each class selected for the experiments of the original training set of (Jeanray et al., 2015)

**FIGURE 3:**
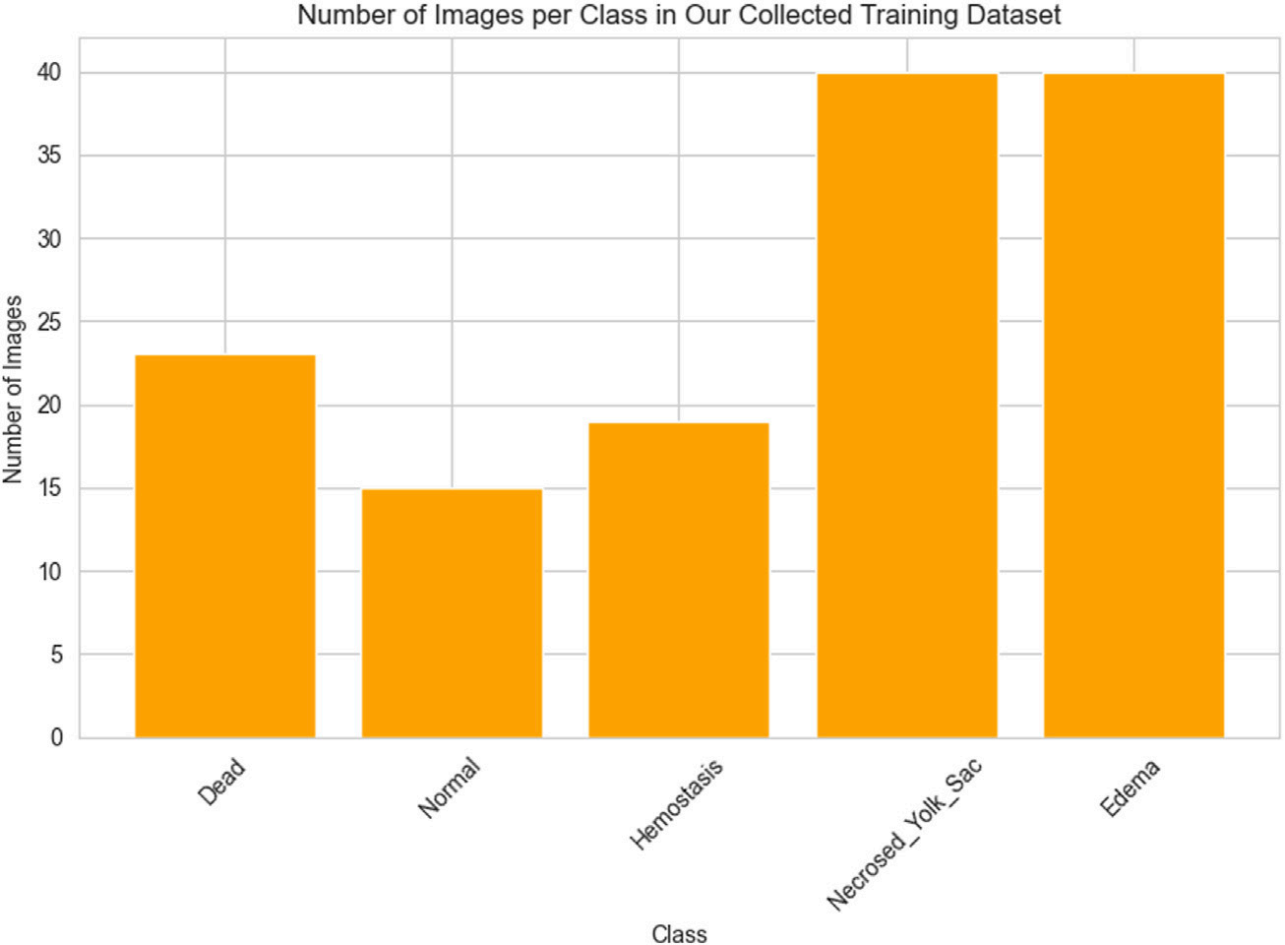
The number of images in each class of our collected dataset.

As previously outlined, the network’s training process employed the dataset provided by Jeanray et al. (2015) and our own collection of images, which were divided into training (learning) and testing segments. As mentioned earlier, from the training images categorized into ten classes by Jeanray et al., we selected images from only five classes. Consequently, the training data comprises a total of 658 images obtained by us from Jeanray et al. (2015) and our collected 137 images. The histograms presented in Figures 2, 3 respectively depict the count of images belonging to each class for the selected dataset of Jeanray et al. (2015) and the training dataset of our collected images.

To evaluate the model’s performance, we additionally collected 96 images from the experiments with QY dosing. Each image is assigned a decision class (representing the final embryo state, such as Edema or Blood stasis) that is utilized in assessing the classification accuracy of the developed model.

#### 2.3.5 Code and computer specifications

The Python programming language was employed in the development of this project, leveraging the extensive ecosystem of data manipulation tools and libraries available within it. The machine learning model development process was orchestrated using Keras, the widely adopted machine learning framework. Keras empowers users to construct sophisticated deep neural networks that incorporate transfer learning and attention mechanisms. All the code used in this project is available at: https://github.com/piotr-maciag/nns_toxic_add.

## 3 Results

### 3.1 Zebrafish experiments

#### 3.1.1 Body width in lateral position of zebrafish embryos

**FIGURE 4:**
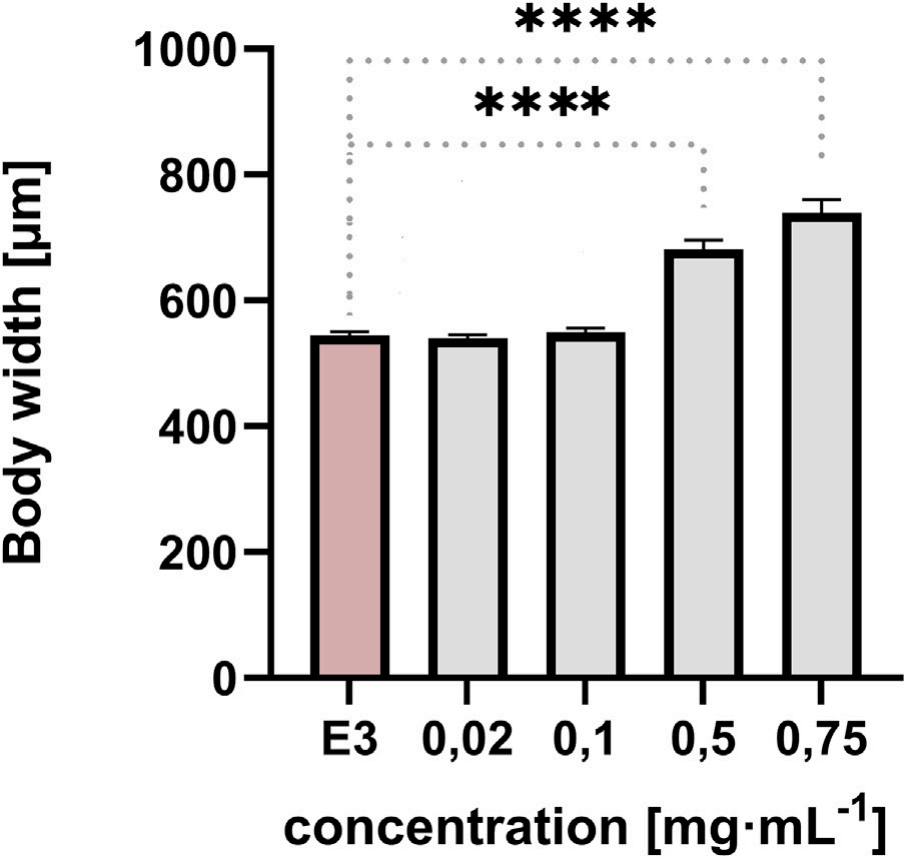
Effect of exposure of embryos to concentrations of QY in the range 0.02–0.75 mg⋅mL^−1^ on the body width of *Danio rerio* [µm], E3 - negative control, **** significantly different from E3 (p < 0.0001), nonparametric Kruskal-Wallis test, ±SEM, n = 20.

**FIGURE 5:**
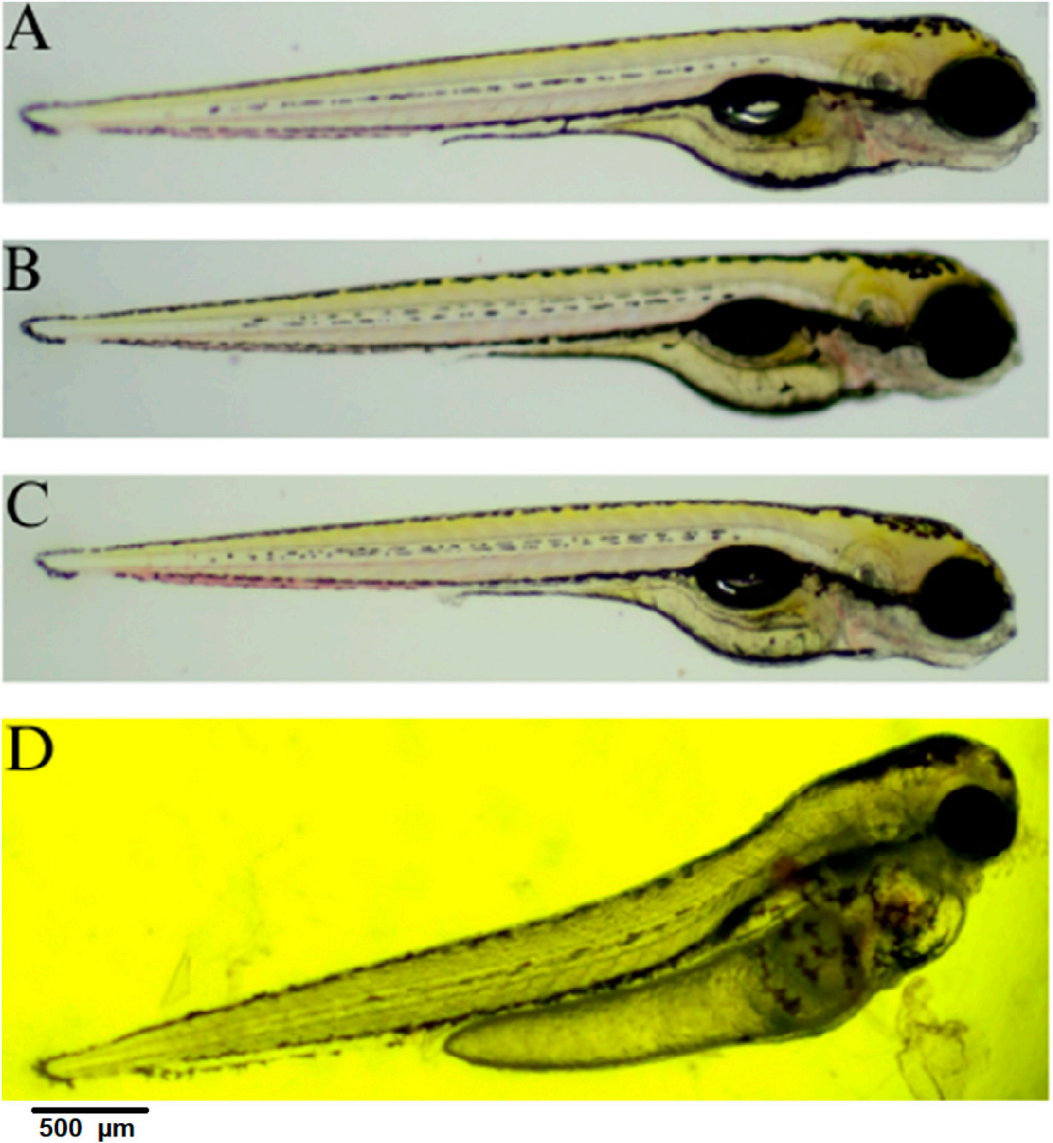
Embryo in lateral position at 96 hpf; **(A)** control E3; **(B)** QY - 0.02 mg⋅mL^−1^; **(C)** QY -0.1  mg⋅mL^−1^; **(D)** QY - 0.5 mg⋅mL^−1^.

The width of the embryo body in a lateral position was quantified at 96 hpf. The measurement was taken from the dorsal strut to the end of the abdomen to ensure repeatability and illustrate the yolk sac swelling (Figures 4, 5). The resulting data were subjected to statistical analysis. A statistically significant difference was observed between the control group and the group exposed to QY concentrations of 0.5 mg⋅mL^−1^, and 0.75 mg⋅mL^−1^ (p < 0.0001) (Figure 4).

#### 3.1.2 Blood stasis

**FIGURE 6:**
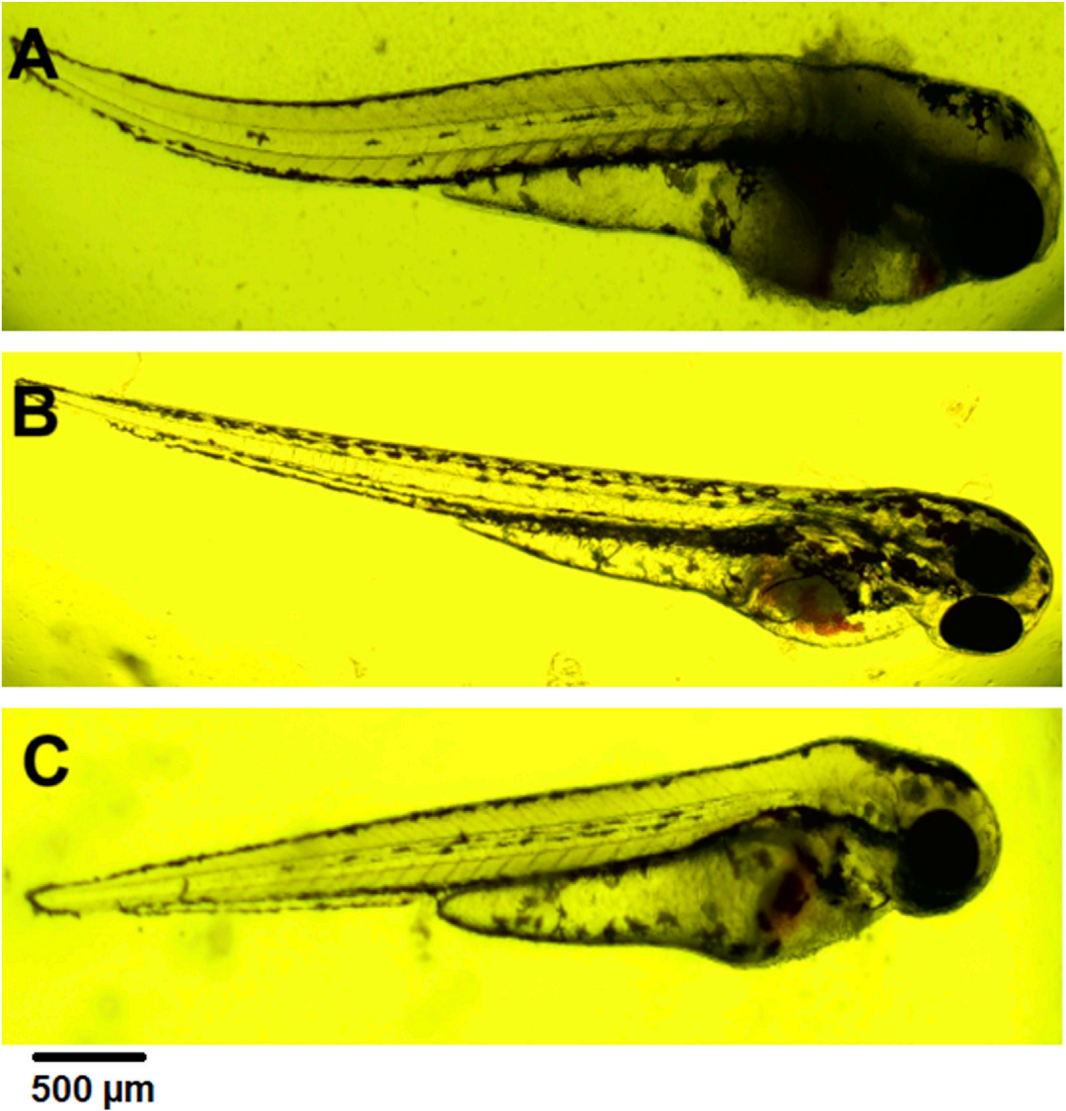
**(A)** coagulation at 72 hpf (2 mg⋅mL^−1^), **(B)** blood stasis at 72 hpf (0.5 mg⋅mL^−1^), **(C)** blood stasis, swollen and necrosed yolk sac, pericardial edema at 96 hpf (0.5 mg⋅mL^−1^).

Blood stasis was an interesting parameter observed during the 72-h experiment (Figures 6B,C). This appeared in different parts of the larvae’s bodies. They usually accompany a lack of circulation while preserving cardiac activity.

#### 3.1.3 Pericardial edema

Pericardial edema was observed at 72 hpf. Its concentration ranged from 0.5 to 2 mg⋅mL^−1^. In each case, it was accompanied by swollen and necrosed yolk sac edema (Figure 6C). These changes were not observed in controls and concentrations of 0.02 mg mL^-1^ and 0.1 mg mL^-1^.

#### 3.1.4 Mortality rate in zebrafish embryo

**FIGURE 7:**
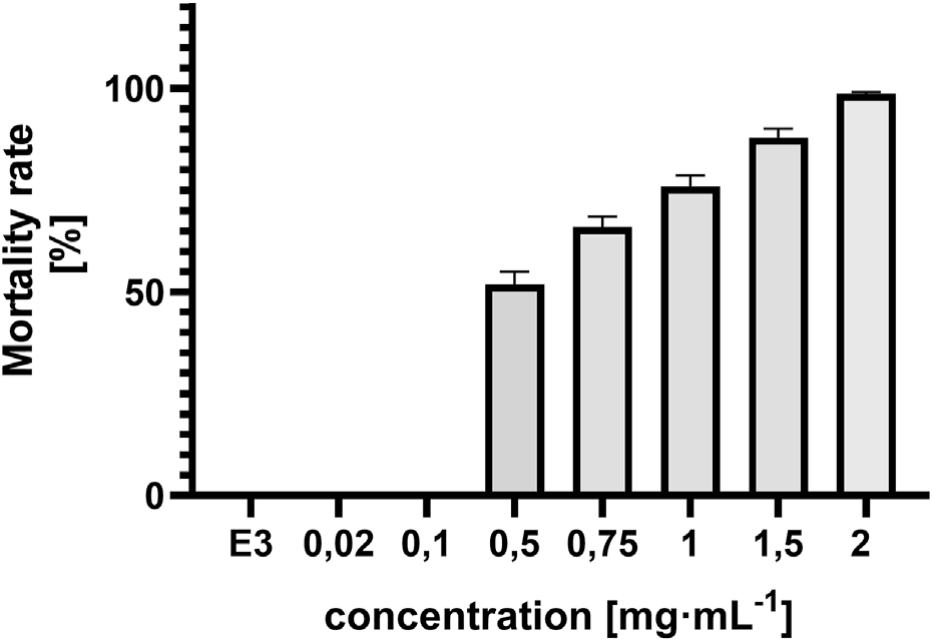
Concentration-dependent mortality of *Danio rerio* at 96 hpf and E3 (negative control), ±SEM, n = 20.

**FIGURE 8:**
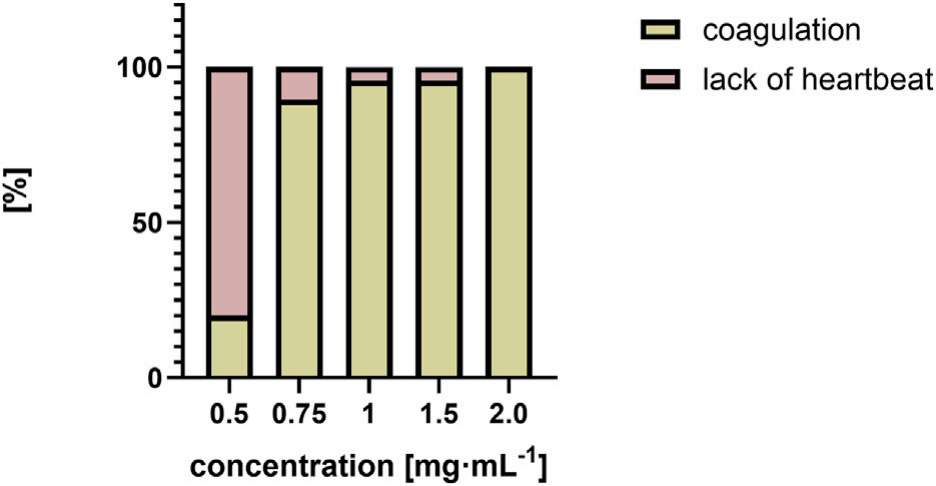
Comparison of causes of death as a percentage [%] over a range of QY concentrations from 0.5 mg⋅mL^−1^ to 2 mg⋅mL^−1^.

Lethal effects were observed, namely, coagulation and absence of heartbeat which were associated with concentrations exceeding 0.5 mg⋅mL^−1^. Coagulation was typically observed as early as 72 hpf (Figure 6A), whereas cessation of the heartbeat was only apparent at 96 hpf, often following circulatory collapse observed at 72 hpf. In the control group, mortality was 0%, while in all groups exposed to the substance at concentrations of 0.5 mg/mL and above, it increased, exceeding 50% at concentrations higher than 0.75 mg mL^-1^ (Figure 7). In addition, a difference in cause of death can be observed in the concentration range 0.5 mg⋅mL^−1^ and above 2 mg⋅mL^−1^ (Figure 8).

#### 3.1.5 Eye size on zebrafish embryos

**FIGURE 9:**
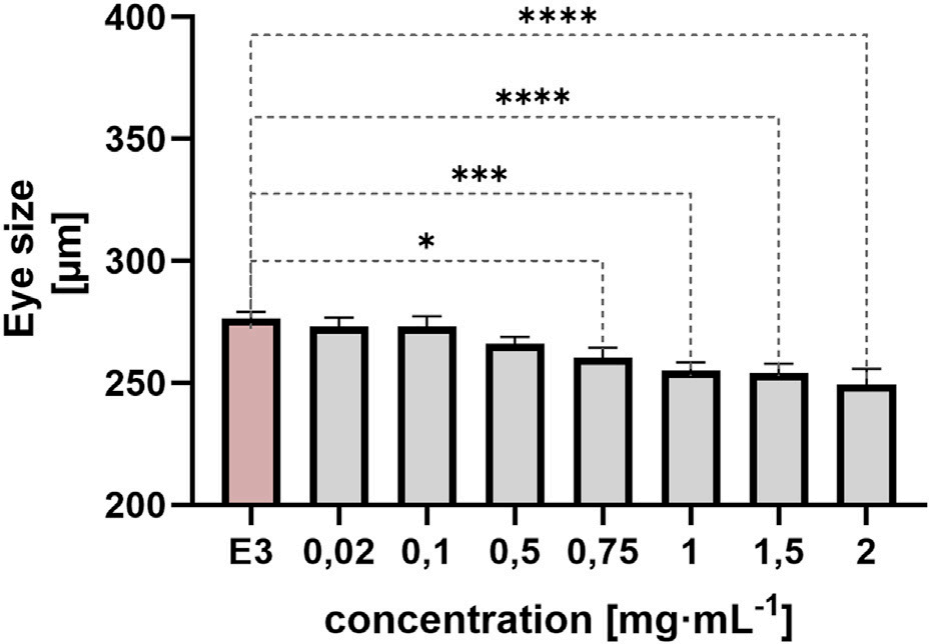
Effect of embryo exposure to QY concentrations in the range 0.02–2 mg⋅mL^−1^ on *Danio rerio* eye size [µm], E3 - negative control, statistically significant differences *p < 0.05, ***p < 0.001, ****p < 0.0001, (Welsh’s ANOVA, [Dunnett’s *post hoc* test]), ±SEM, n = 20.

Eye size was measured at 72 hpf. The following day, measurement at 96 hpf could not be performed due to numerous coagulations. In addition to more general phenotypes, QY also caused microphthalmia (small eyes) (Figure 9). A statistically significant difference was observed between the control group and the groups exposed to QY at concentrations of 0.75 mg/mL (*p < 0.05), 1 mg/mL and 1.5 mg/mL (***p < 0.001), and 2 mg/mL (**p < 0.0001).

#### 3.1.6 Calculation of LC50

**FIGURE 10:**
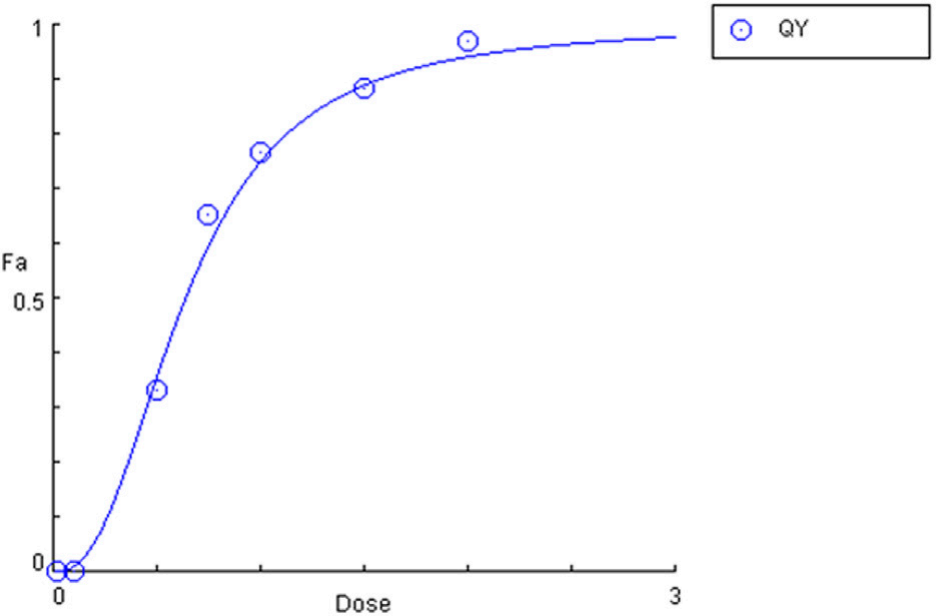
Plot of the dependence of mortality (Fa) on quinoline yellow (QY) concentration (dose).

Given that the mortality rate exceeded 50%, it was decided that the lethal concentration value (LC50) should be calculated using the probit method. A straight line was plotted after converting the concentration to a logarithmic value and fitting the probit to the mortality value. The LC50 value was then calculated from the equation of the straight line for probit 5 (y = 5), which indicates a 50% mortality rate (LC50 = 0,64 mg⋅mL^−1^). To confirm the LC50 result obtained by the probit method, an analysis was conducted in the CompuSyn software (CompuSyn Inc., Paramus, US). The calculated value in the program was also 0.64 mg⋅mL^−1^ (Figure 10).

### 3.2 Prediction of quinoline yellow toxicity using *in silico* studies ADMET predictor

A toxicity study was initiated with an analysis conducted using ADMET Predictor, a machine learning platform designed explicitly for ADMET modelling. This platform is equipped with advanced data analysis capabilities and compound metabolism prediction functionalities (https://www.simulations-plus.com/software/admetpredictor/toxicity/) (Toxicity Module, 2025). Furthermore, ADMET Predictor is a predictive tool for detecting human toxicity parameters, including carcinogenic, cardiotoxic, and hepatotoxic effects. It enables the prediction of developmental toxicity and mutagenicity of compounds. *In silico* studies, a high probability of QY inducing skin and respiratory sensitisation was demonstrated in a rat and mouse model. Based on its structural alerts and physicochemical properties, the compound QY is classified as toxic, in accordance with established predictive toxicology frameworks. The murine Local Lymph Node Assay (LLNA), a validated and reproducible method for assessing the relative potency of chemical skin sensitizers, was employed in the present study. Additionally, a qualitative assessment of respiratory sensitization potential was conducted using a rat model, with the results summarized in Table 1. Reproductive toxicity was evaluated using standardized endpoints and is also presented in Table 1. The term ‘reproductive toxicity’ refers to any factor that disrupts an organism’s reproductive capabilities. This encompasses a range of adverse effects, including damage to reproductive organs, behavioural changes, infertility and impaired offspring development, both during and after gestation. In this study, the ADMET Predictor utilised data from the FDA/TETRIS database, which was initially sourced from the literature. Additionally, clastogenicity and mutagenicity (MUT) studies were conducted, based on the calculation of chromosomal aberrations (Chrom_Aberr) and the prediction of Ames test results (Table 1).

**TABLE 1:** Predicted toxicities for QY compounds performed by ADMET Predictor software.

Sens_Skin	Sens_Resp	Repro_Tox	Hepatotoxicity					Mutagenity		Chrom_Aberr	Estro_Filter	Andro_Filter
			Ser_AlkPhos	Ser_GGT	Ser_LDH	Ser_AST	Ser_ALT	MUT_97 + 1537	MUT m97 + 1537; MUT m98; MUT m100; MUT m102+wp2 and MUT m1535; MUT 98; MUT 100; MUT 102+wp2, MUT 1535			
S	S	T	NL	EL	NL	NL	NL	P	N	T	T	T

abbreviations: Sens_Skin - skin sensitivity, Respiratory sensitivity (Sens_Resp), Reproductive toxicity (Repro_Tox), Hepatotoxicity (Ser_AlkPhos, Ser_GGT, Ser_LDH, Ser_AST, Ser_ALT), Mutagenity (MUT), Chromosome aberration (Chrom_Aberr), Estrogen Toxicity (Estro_Filter) and Androgen Toxicity (Andro_Filter) quantify affinity for estrogen and androgen receptors, S - sensitive, EL, elevated; NL, normal, T–toxic, NT, nontoxic, P–positive, N - negative.

The mutagenicity models were derived from the Carcinogenic Chemicals Database (CPDB). The ten models (MUTs) were employed individually to assess the predicted mutagenicity of five *Salmonella typhimurium* strains with microsomal activation (MUT m97 + 1537; MUT m98; MUT m100; MUT m102+wp2 and MUT m1535) and without microsomal activation (MUT 97 + 1537; MUT 98; MUT 100; MUT 102+wp2 and MUT 1535). QY showed predictable mutagenicity only for *S. typhimurium* strains TA97 and/or TA1537, while it was not found for other strains (Table 1). An artificial neural network ensemble model named provided by ADMET Predictor is used to assess the clastogenic potential of QY (Table 1). The parameters related to liver toxicity were found to be within an acceptable range, with no discernible impact on the activity of alkyl phosphatase (AlkPhos), lactate dehydrogenase (LDH), aspartate transaminase (AST) and alanine transaminase (ALT). The results of the analyses conducted in ADMET Predictor indicate that QY may induce an increase in γ-glutamyl transferase activity (GGT) (Table 1). Another parameter was related to endocrine disruption. The objective was to ascertain whether the molecule would exhibit a discernible affinity for the estrogen receptor through utilising two neural network models. A toxic result indicates the presence of a detectable affinity for the receptor. The Estro_Filter model is a second model that predicts the degree of binding of a compound to the oestrogen receptor. Similarly, a neural network model (Andro_Filter) for the androgen receptor was developed. Qualitative estimates of androgen and oestrogen receptor toxicity in rats for QY are presented in Table 1. The model analysing the structure of QY predicts that it can compete with sex hormones to inhibit and interact with oestrogen and/or androgen receptors, potentially disrupting endocrine system signalling. This disruption can block the normal flow of hormone signals and lead to toxicity (Table 1).

### 3.3 Results of image classification with designed neural network’s architecture

The designed neural network model was evaluated in the experiments described below. Firstly, we describe the learning parameters used in the model’s training process. The learning parameters specified in Table 2 were determined through meticulous preliminary experiments. Specifically, each training dataset was partitioned into actual training and validation sets in an 8:2 ratio. During each training phase, the batch size was set to 16 images. Early stopping was implemented in every training phase, with the maximum number of training epochs set to 100. The initial learning rate was progressively reduced during the training phases. The early stopping condition was configured to reduce the learning rate by a factor of 0.5 if the validation loss did not exhibit an improvement for five consecutive epochs, with a minimum learning rate capped at 1e-6.

**TABLE 2:** Basic learning parameters of designed neural Network’s architecture different training phases.

Parameter	First phase (initial training)	Second phase (fine-tuning)	Third phase (fine-tuning on new data)
Data Used	Jeanray-2015 Selected Training Data	Jeanray-2015 Selected Training Data	Our training data
Base Model	ResNet50 (ImageNet weights network’s)	Same as First Phase	Same as First Phase
Attention Mechanism	CBAM applied to base model output	Same as First Phase	Same as First Phase
Frozen Layers	All base model layers	First 1/3 of base model layers frozen	None; all layers are trainable
Unfrozen Layers	Only new layers added on top	Last 2/3 of base model layers and new layers	Entire model is trainable
Optimizer	Adam optimizer	Adam optimizer	Adam optimizer
Initial Learning Rate	1e-4	1e-5	1e-5
Batch Size	16	16	16
Epochs	Up to 100 (with early stopping)	Up to 100 (with early stopping)	Up to 100 (with early stopping)
Loss Function	Categorical Crossentropy	Categorical Crossentropy	Categorical Crossentropy
Metrics	Accuracy	Accuracy	Accuracy

To evaluate the performance of the designed architecture, we employed several metrics. The final condition of an embryo can often be described by multiple states (e.g., Edema and Blood Stasis). In multi-class classification, the model frequently outputs more than one decision class along with their associated probabilities. To assess classification quality, we utilized the concepts of Top-1, Top-2, and Top-3 predicted classes:

• Top-1 evaluation selects the class with the highest predicted probability as the model’s output for evaluation.
• Top-2 and Top-3 evaluations determine a prediction as correct if the actual class is among the top 2 or top 3 predicted classes, respectively.

**FIGURE 11:**
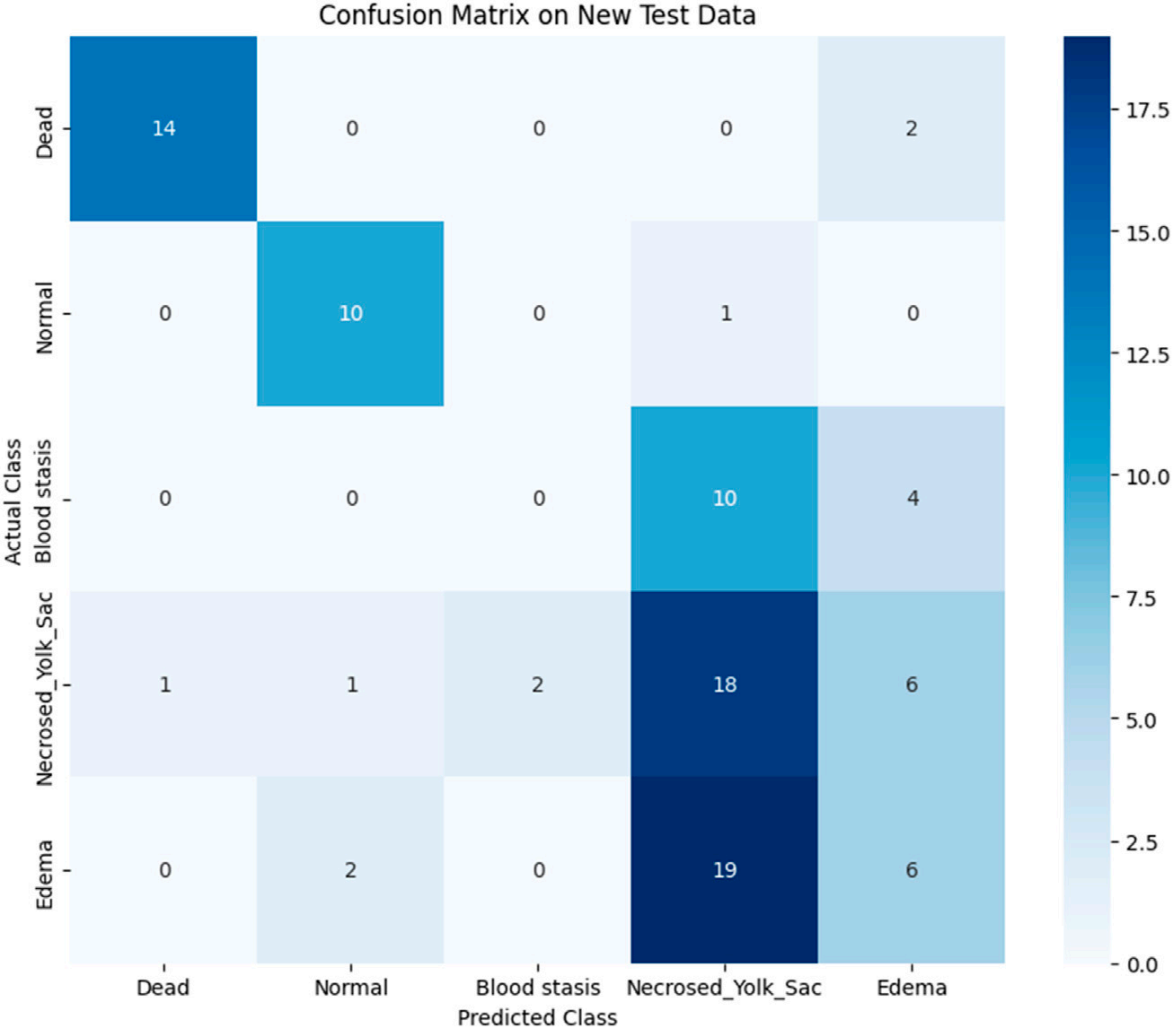
Confusion matrix for Top-1 evaluation.

**FIGURE 12:**
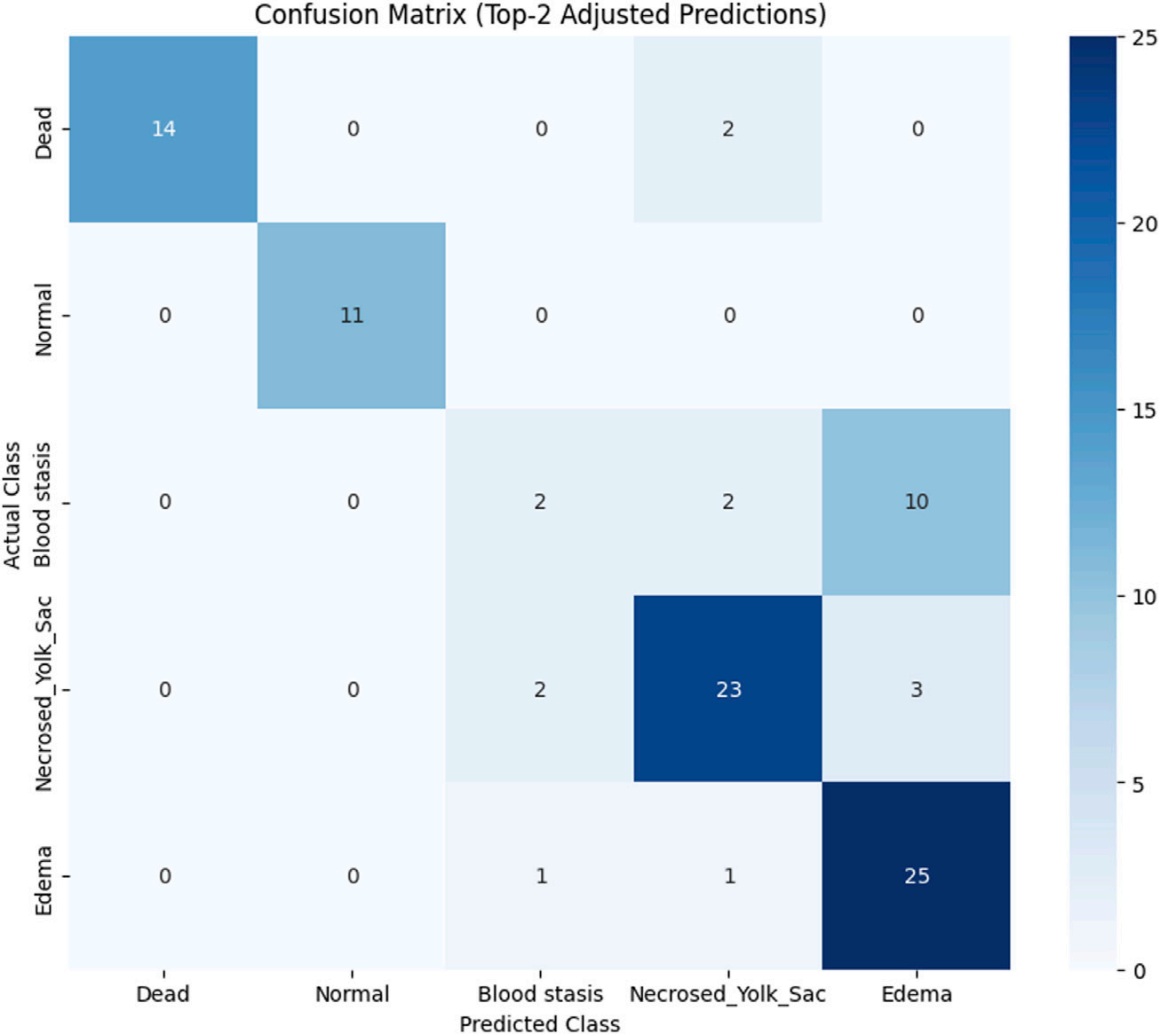
Confusion matrix for Top-2 evaluation.

**FIGURE 13:**
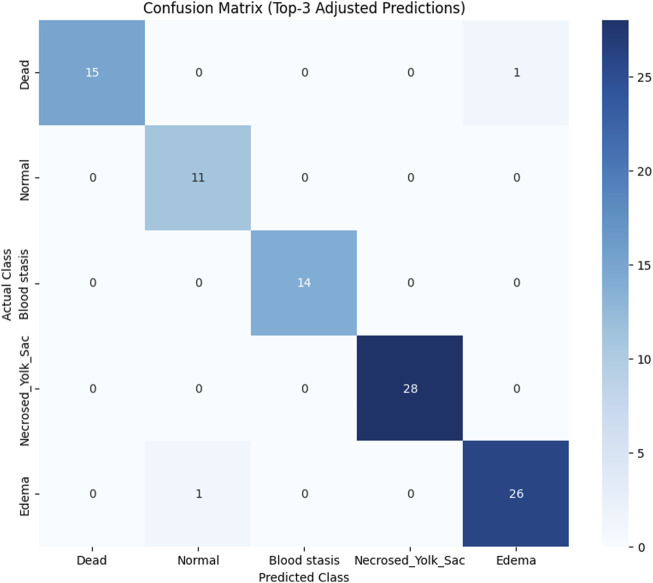
Confusion matrix for Top-3 evaluation.

To assess the classification performance, we employed four metrics: Precision, Recall, Accuracy, and F1 Score. The respective formulas are presented below. These metrics are reported separately for evaluations of the Top-1, Top-2, and Top-3 predictions for each base model (Tables 3–5). As can be noted from the tables, the ResNet50 based model tends to provide the best classification results. Furthermore, confusion matrices are presented for the Top-1, Top-2, and Top-3 evaluations for the ResNet50 base model, which yielded the most favorable classification outcomes (Figures 11–13).

$$Precision=\frac{True\;Positives}{True\;Positives+False\;Positives}$$

$$Recall=\frac{True\;Positivies}{True\;Positives+False\;Negatives}$$

$$Accuracy=\frac{True\;Positives+True\;Negatives}{Total\;Number\;of\;Images\;in\;Test\;Set}$$

$$F1=2\times \frac{Precision\times Recall}{Precision+Recall}$$

**TABLE 3:** Classification results for the Top-1 evaluation of predictions.

	ResNet50 base model			VGG16 base model			Xception base model		
Class	Precision	Recall	F1-Score	Precision	Recall	F1-Score	Precision	Recall	F1-Score
Dead	0.93	0.88	0.90	0.68	0.81	0.74	0.17	1	0.29
Normal	0.77	0.91	0.83	0.80	0.73	0.76	0.00	0.00	0.00
Blood stasis	0.00	0.00	0.00	0.00	0.00	0.00	0.00	0.00	0.00
Necrosed_Yolk_Sac	0.38	0.64	0.47	0.32	0.29	0.30	0.00	0.00	0.00
Edema	0.33	0.22	0.27	0.31	0.48	0.38	0.00	0.00	0.00
Average	**0.48**	**0.53**	**0.50**	**0.42**	**0.46**	**0.44**	**0.03**	**0.20**	**0.06**
Accuracy	**0.50**			**0.44**			**0.17**		

Bold values in the tables indicate the best performance.

**TABLE 4:** Classification results for the Top-2 evaluation of predictions.

	ResNet50 base model			VGG16 base model			Xception base model		
Class	Precision	Recall	F1-Score	Precision	Recall	F1-Score	Precision	Recall	F1-Score
Dead	1.00	0.88	0.93	0.93	0.88	0.90	1.00	1.00	1.00
Normal	1.00	1.00	1.00	1.00	0.73	0.84	0.00	0.00	0.00
Blood stasis	0.40	0.14	0.21	0.00	0.00	0.00	0.00	0.00	0.00
Necrosed_Yolk_Sac	0.82	0.82	0.82	0.66	0.96	0.78	0.37	0.68	0.48
Edema	0.66	0.93	0.77	0.78	0.93	0.85	0.38	0.41	0.39
Average	**0.78**	**0.75**	**0.75**	**0.67**	**0.79**	**0.68**	**0.35**	**0.42**	**0.37**
Accuracy	**0.78**			**0.77**			**0.48**		

Bold values in the tables indicate the best performance.

**TABLE 5:** Classification results for the Top-3 evaluation of predictions.

	ResNet50 base model			VGG16 base model			Xception base model		
Class	Precision	Recall	F1-Score	Precision	Recall	F1-Score	Precision	Recall	F1-Score
Dead	1.00	0.94	0.97	0.94	0.94	0.94	0.39	1.00	0.56
Normal	0.92	1.00	0.96	1.00	0.91	0.95	0.00	0.00	0.00
Blood stasis	1.00	1.00	1.00	1.00	0.86	0.92	0.00	0.00	0.00
Necrosed_Yolk_Sac	1.00	1.00	1.00	0.93	1.00	0.97	1.00	1.00	1.00
Edema	0.96	0.96	0.96	0.96	1.00	0.98	1.00	1.00	1.00
Average	**0.98**	**0.98**	**0.98**	**0.97**	**0.94**	**0.95**	**0.48**	**0.60**	**0.51**
Accuracy	**0.98**			**0.96**			**0.74**		

Bold values in the tables indicate the best performance.

**FIGURE 14:**
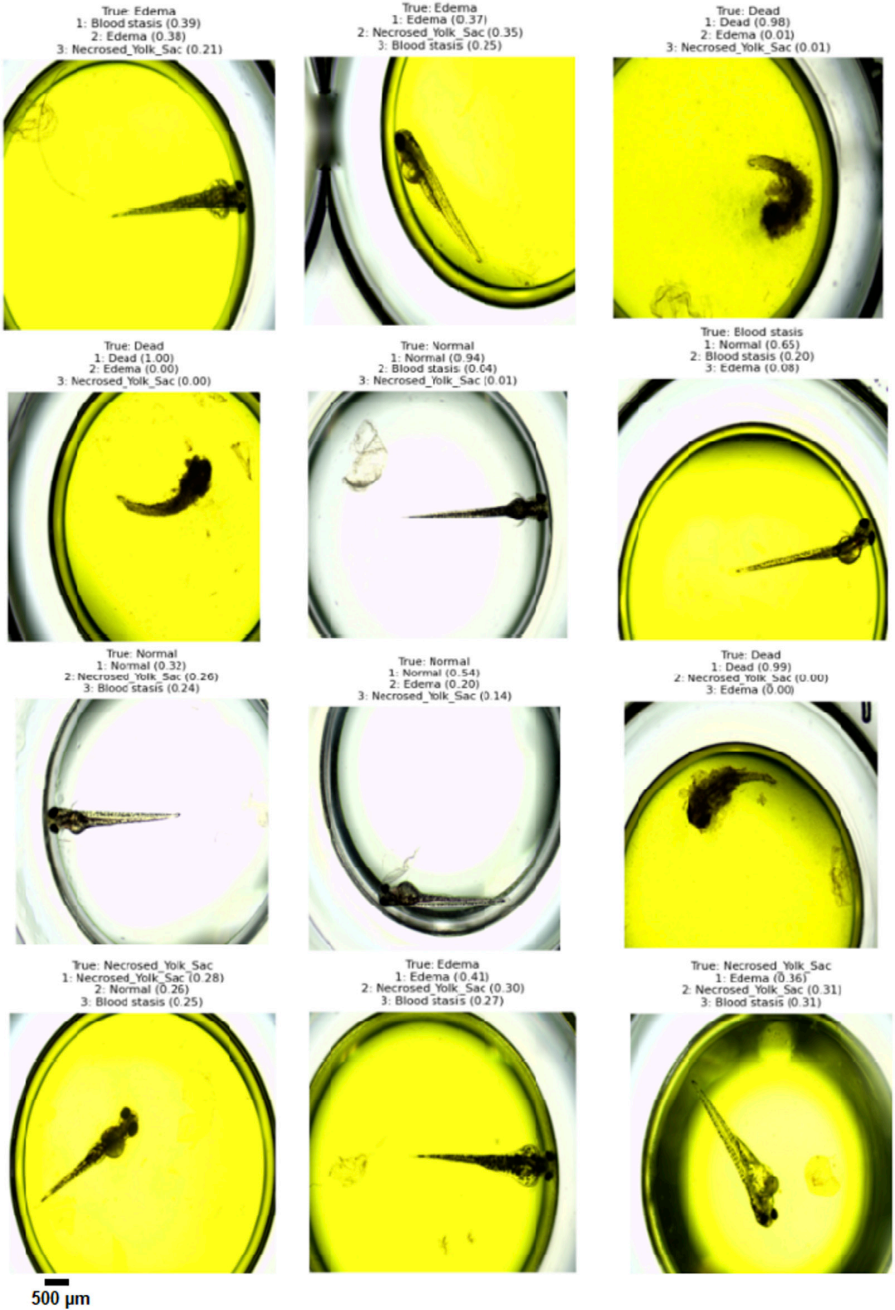
Example Top-3 predictions with the designed model (the ResNet50 base model was ued) on randomly selected images from test dataset.

The results demonstrate that the proposed model (especially when the ResNet50 based model was used) achieves very strong classification performance for the *Dead* and *Normal* decision classes across all three evaluation types. For the classes *Blood stasis*, *Necrosed Yolk Sac*, and *Edema*, the model performs significantly better in the Top-2 and Top-3 evaluations than in Top-1 results. This can be attributed to the model’s tendency to consider multiple states as applicable, which aligns with the inherent overlap in these conditions. Figures 11–13 illustrate the confusion matrices for the Top-1, Top-2, and Top-3 evaluations using the ResNet50 based model, respectively. The examples of predictions of classes with the model are shown in Figure 14.

## 4 Discussion

*In silico* predictions based on the structures of QY indicated that the compound may cause endocrine disruption, chromosomal aberrations, reproductive toxicity and mutagenic effects in the Ames test, as well as skin and respiratory sensitisation in animal models. Furthermore, no hepatotoxic effects were observed, although elevated γ-glutamyl transferase levels were noted in the presence of QY. In a study conducted by Damayanti et al. (2015), the researchers employed *in silico* methods to predict the acute toxicity (LD50), mutagenicity, carcinogenicity, reproductive toxicity, chronic toxicity (NOEL), metabolite toxicity for synthetic additives that act as antioxidants. In a separate study, butylhydroxytoluene (BHT) was employed as a case study to examine the efficacy of computational techniques, including reverse screening and molecular docking, in identifying protein-ligand interactions of artificial additives based on their toxicological effects (Tortosa et al., 2020). *In silico* analyses can be employed to ascertain the characteristics of synthetic additives and to examine their functional mechanisms or potential adverse effects. It is worthy of note that this may be relevant in cases where experimental studies are clearly lacking, as is the case with BHT. In the search for xenoestrogens in synthetic additives, an integrated *in silico* and *in vitro* approach has been employed, as outlined in (Amadasi et al., 2009). In recent years, there has been a notable increase in the number of studies examining the toxic effects of food additives, with the brindle danio serving as a model organism in many of these investigations. The toxic effects of food additives in the zebrafish model have been found to be primarily manifested as dose-dependent developmental toxicity. The findings of the Sarmah et al. (2020) analysis demonstrate the developmental toxicity of the synthetic antioxidant BHT. Other studies employing the zebrafish model have indicated that exposure to sodium dehydroacetate (DHA-S), an approved preservative commonly added to processed foods, may represent a potential cardiovascular risk factor. In a comprehensive evaluation of the adverse effects of propylparaben and methylparaben on the early developmental stages of Bereketoglu and Pradhan (2019) observed a range of abnormalities, including spine and pigmentation defects, pericardial edema and reproductive toxicity. Furthermore, the altered expression of the androgen receptor (AR) and estrogen receptor 2 alpha (ESR2a) indicated anti-androgenic and estrogenic effects of parabens in zebrafish.

Synthetic dyes are frequently employed as colourings, particularly in confectionery products. Consequently, it can be hypothesised that they may have more adverse health effects in children than in adults. In view of the toxicity of azo dyes, EU countries regularly undertake reviews and revisions of their ADI values. The developmental toxicity of four azo dyes, including Tartrazine, Sunset Yellow, Amaranth Red and Allura Red, has been evaluated using zebrafish embryos. At concentration levels of 5–50 mM, it has been demonstrated that azo dyes can impede the process of leaving the chorion and induce developmental abnormalities, including cardiac edema, a slowed heart rate, a swollen yolk follicle, a curvature of the spine and a tail deformity. The embryos demonstrated complete lethality at 100 mM of the analysed azo dyes. Conversely, impediments to the embryos’ departure from the chorion and aberrant developmental outcomes were observed at concentrations exceeding the ADI (Jiang et al., 2020). The dose-dependent toxic effects of the caramel colouring agent E150d on the embryos of zebrafish included disruption of chorion exit, survival, phenotype, heartbeat, and swimming ability, as well as damage to skeletal muscle and the pericardial cavity. These effects were observed at varying doses of the food dye sulphite ammonia caramel (Capriello et al., 2021; Hou et al., 2023).

The findings of the present study indicate that QY induces lethal alterations in embryos of the zebrafish (*Danio rerio*). The two endpoints observed at the 96-h exposure period were coagulation and the absence of a heartbeat. Embryos exposed to a QY solution at concentrations of 0.1 mg⋅mL^−1^ and above exhibited a higher mortality rate. At a concentration of 0.5 mg⋅mL^−1^ mg, the survival rate was 46.47% for embryos. The LC50 parameter value was found to be 0.64 mg⋅mL^−1^. In a study conducted by Duy-Thanh et al. (2022), the embryotoxic and teratogenic effects of QY were analysed using a zebrafish model. The mortality data yielded divergent results from those of the present study. The survival rate remained above 90% up to a concentration of 0.5 mg⋅mL^−1^. The authors of the publication report that the median lethal concentration level was 6.89 mg⋅mL^−1^. Joshi and Katti (2018) conducted embryotoxicity studies using the zebrafish model to assess the toxicity of Tartrazine, a compound belonging to the azo-structured chemical class. An LC50 value of 15.7 mg⋅mL^−1^ was determined. Moreover, the aforementioned authors conducted a similar study, this time focusing on orange yellow. The LC50 value was approximately 19.3 mg⋅mL^−1^. The substance under examination in this study displays a greater toxicity potential than other azo dyes. Among the embryotoxic effects observed, developmental defects such as yolk sac swelling and reduced eye size were noted. These alterations were observed at concentrations of 0.5 mg⋅mL^−1^ and 0.75 mg⋅mL^−1^, respectively. The test dye was observed to contribute to the development of cardiac emphysema and blood stasis, while maintaining circulation and cardiac activity. In the aforementioned study, standard substances devoid of impurities were subjected to analysis. The occurrence of yolk sac edema was observed in over 50% of the larvae at concentrations of 0.5 mg⋅mL^−1^. Conversely, isolated instances of under-eye were documented at concentrations as low as 0.02 mg⋅mL^−1^, with this phenotype occurring in nearly 100% of larvae at concentrations of 0.5 mg⋅mL^−1^. The swelling of the yolk sac may be associated with impaired absorption of the nutrients it contains (Ramos‐Souza et al., 2023; Kashyap et al., 2007; (Harding et al., 2021). Our study identified a form of blood stasis that has not previously been described in the scientific literature. A recent transcriptomics study has demonstrated that exposure to a non-sulphonated form of QY results in a reduction in the expression of metabolic genes in zebrafish embryos. Of particular note is the disruption of the retinoic acid signalling pathway, which suggests a potential impairment in eye development. The observation that a widely used food additive may interfere with nutrient metabolism, even at sub-ADI exposure levels, warrants a critical re-evaluation of its current safety thresholds (0.5 and 3 mg/kg b.w., respectively, set by EFSA and JECFA) (Refined exposure assessment for quinoline yellow, 2015). Further studies are required to elucidate the toxicity of synthetic additives using the model organism *Danio rerio*. A paucity of definitive experimental data exists in the global scientific literature on the safety of chemical additives, including their interaction effects during the embryonic development of the organism. Recent studies indicate that quinoline yellow can induce protein aggregation (Khan et al., 2019). One *in vitro* study demonstrated that the dye may modulate the expression of 21 genes involved in DNA repair, raising concerns about its toxicological implications (Chequer et al., 2015). Additionally, quinoline yellow acts as a potent agonist of the aryl hydrocarbon receptor (AHR), induces CYP1A1 expression, and inhibits estrogen receptor signaling through AHR-dependent pathways, suggesting potential endocrine-disrupting effects (Tarnow et al., 2020)

The application of machine learning to replace manual measurements would undoubtedly enhance the applicability and efficiency of zebrafish model. Machine learning approaches are becoming increasingly widespread and are now present in most areas of research. CNN employ the concept of deep learning, wherein the augmentation of the number of hidden neuron layers within the network leads to a concomitant enhancement of overall image recognition accuracy. To further augment image recognition capabilities with CNNs, techniques such as transfer learning and attention mechanisms can be employed. In transfer learning, a pretrained recognition model (for instance, an ImageNet CNN trained on a comprehensive data set of images) is selected and augmented with additional layers or modified to adopt a distinct model architecture. This approach is particularly beneficial in scenarios where the availability of domain-specific training data is constrained, such as in the classification of biomedical images. Kim et al. (2022) reviewed 121 publications on the application of transfer learning in medical image recognition. The study outlines various transfer learning approaches, where a pretrained CNN can be extended with additional convolutional layers or used as a feature extractor, with the extracted features then applied to train a separate machine learning model, such as a Support Vector Machine (SVM). Additionally, the pretrained model may or may not be fine-tuned with new data. As indicated by Kim et al. (2022), the pretrained ResNet50 CNN model achieved superior classification results compared to two other models, AlexNet and VGG, when trained on the ImageNet dataset. As presented by our experiments, the base ResNet50 model also provides the best classification results.

Additionally, to augment classification accuracy, we incorporated the attention mechanism into CNNs. The attention mechanism in machine learning emulates the human capacity to concentrate on specific portions of information to enhance comprehension. Tsotsos et al. (1995) and Niu et al. (2021) identify two types of human attention. The first type of attention is often induced by a stronger stimulus that draws human focus, while the second involves intentional concentration on a task. In general, machine learning implements the latter type of attention mechanism. Previous research has indicated that applying an attention mechanism can improve the quality of an image recognition task (Rodriguez et al., 2020). Specifically, in this work we decided to adapt the Convolutional Block Attention Mechanism (CBAM). This attention mechanism comprises two modules: the Channel Attention Module and the Spatial Attention Module. As outlined by the authors of (Woo et al., 2018), CBAM has been demonstrated to enhance image classification outcomes. In our research, we applied CBAM to improve the detection of abnormal blood stasis in embryos. Blood stasis is frequently distinguished from other pathological states by the presence of minute blood clots in the images. However, our experiments indicated that a neural model lacking an attention mechanism encountered difficulties in accurately identifying these blood clots. In summary, we posit that the developed model can effectively support the identification of pathological states in *Danio rerio* embryos induced by specific concentrations of QY. The results suggest that these concentrations of QY may indeed have deleterious effects.

## 5 Conclusion

The *in silico* study presents evidence suggesting that QY may pose potential health risks, including developmental toxicity, allergic activity, and possible endocrine disruption. These findings indicate that the current safety assessments may be insufficient and warrant a more rigorous evaluation of QY, potentially accompanied by stricter regulatory measures. The synthetic colour QY, has been observed to induce lethal changes in *Danio rerio* embryos, at concentrations ranging from 0.5 mg⋅mL^−1^ to 2 mg⋅mL^−1^. The lethality of *Danio rerio* is evidenced by the formation of cardiac dysfunction and coagulation. The LC50 of QY was determined to be 0.64 mg⋅mL^−1^. The administration of QY at concentrations above 0.5 mg⋅mL^−1^ has been observed to elicit a dose-dependent pericardial edema, swollen and necrosed yolk sac, blood stasis and reduced eye size. Attention-based CNNs, combined with transfer learning, can be employed for the classification of developmental toxicity in zebrafish embryos. The model demonstrated consistent performance in identifying healthy samples but exhibited challenges in distinguishing between disease states. Notably, the applied AI model also suggests that the employed doses of QY induce pathological states in embryos. The findings of these studies on the embryotoxic potential of QY underscore the necessity for further experiments to gain a comprehensive understanding of the health effects of this dye, particularly within the context of embryonic development. Consequently, further research is required to provide consistent data, identify suitable alternatives to chemical colours, and assess their safety.

## Data Availability

The raw data supporting the conclusions of this article will be made available by the authors, without undue reservation.
